# Language repetition and short-term memory: an integrative framework

**DOI:** 10.3389/fnhum.2013.00357

**Published:** 2013-07-12

**Authors:** Steve Majerus

**Affiliations:** ^1^Department of Psychology - Cognition and Behavior, Université de LiègeLiège, Belgium; ^2^Fund for Scientific Research - FNRSBrussels, Belgium

**Keywords:** language, repetition, short-term memory, working memory, serial order, attention

## Abstract

Short-term maintenance of verbal information is a core factor of language repetition, especially when reproducing multiple or unfamiliar stimuli. Many models of language processing locate the verbal short-term maintenance function in the left posterior superior temporo-parietal area and its connections with the inferior frontal gyrus. However, research in the field of short-term memory has implicated bilateral fronto-parietal networks, involved in attention and serial order processing, as being critical for the maintenance and reproduction of verbal sequences. We present here an integrative framework aimed at bridging research in the language processing and short-term memory fields. This framework considers verbal short-term maintenance as an emergent function resulting from synchronized and integrated activation in dorsal and ventral language processing networks as well as fronto-parietal attention and serial order processing networks. To-be-maintained item representations are temporarily activated in the dorsal and ventral language processing networks, novel phoneme and word serial order information is proposed to be maintained via a right fronto-parietal serial order processing network, and activation in these different networks is proposed to be coordinated and maintained via a left fronto-parietal attention processing network. This framework provides new perspectives for our understanding of information maintenance at the non-word-, word- and sentence-level as well as of verbal maintenance deficits in case of brain injury.

Short-term maintenance processes are a core ingredient of language repetition due to the inevitable temporal separation of input and output processes, implicating a delay period during which the input has to be temporarily maintained, even if often for a very brief time period such as during repetition of short, single word stimuli. However, the relationship between language repetition and maintenance processes remains poorly understood, partly due to the parallel and independent evolution of research in language processing and verbal short-term memory (STM) domains: many cognitive and neural models of language processing remain vague about the nature and neural underpinnings of maintenance processes, and most models of verbal STM, although acknowledging links with the language system, do not consider these links with much detail. In her recent review, Friederici (2012) highlighted the need for language processing architectures to consider and integrate interactions with STM. We here provide a review of studies that have investigated the cognitive and neural networks of maintenance processes and their interaction with language repetition/reproduction processes from various theoretical and methodological perspectives. We will attempt at bridging the gap between language processing and STM architectures, by proposing an integrative framework of verbal maintenance and language processing in which maintenance of verbal information is an emergent process, resulting from the temporary activation of both dorsal and ventral language processing pathways and their interaction with attentional control and sequence representation systems.

## The role of dorsal and ventral pathways in STM maintenance

Recent models of language repetition (Jacquemot and Scott, 2006; Hickok and Poeppel, 2007; Friederici, 2012; Hickok, 2012) assume that the posterior superior temporal gyrus (pSTG) plays a central function during language repetition, by providing, via the dorsal stream of speech processing, a sensorimotor interface linking acoustic codes in the superior temporal gyrus to articulatory codes in the posterior inferior frontal gyrus. This function has been considered to interface input and output phonological representations and to buffer verbal information via the temporary activation of these representations in language repetition tasks (Jacquemot and Scott, 2006; Hickok and Poeppel, 2007). The most compelling evidence supporting the pSTG region as a buffer function comes from patients presenting lesions in the pSTG area and whose language repetition deficit is most parsimoniously explained by difficulties in maintaining verbal information during repetition, such as in different cases of conduction aphasia or of the logopenic variant of primary progressive aphasia (Buchsbaum and D'Esposito, 2008; Gorno-Tempini et al., 2008; Buchsbaum et al., 2011). This is most clearly illustrated by patients with deep dysphasia, a rare but highly compelling form of conduction aphasia. These patients have severe difficulties in repeating single words, with marked lexicality and word imageability effects: repetition of familiar, concrete words is much less impaired than repetition of non-words or low imageability words (Michel and Andreewsky, 1983; Duhamel and Poncet, 1986; Howard and Franklin, 1988; Martin and Saffran, 1992; Trojano et al., 1992; Croot et al., 1999; Majerus et al., 2001; Tree et al., 2001; Wilshire and Fisher, 2004). A further hallmark characteristic is the production of semantic paraphasias during single word repetition. The most parsimonious account that has been proposed to explain this symptom constellation is a phonological decay impairment: activated phonological representations decay at an abnormally accelerated rate, with only some residual semantic activation left at the moment of production, explaining the strong influence of lexical and semantic variables on repetition performance (Martin and Saffran, 1992; Martin et al., 1994a). If abnormally increased decay of phonological representations is the defining feature of this syndrome, then the duration of the delay between input and output stages during language repetition should be a critical variable. This is supported by a case study with a deep dysphasic patient who has partially recovered from his language impairment, but who shows again semantic effects during repetition as soon as the delay between language input and output is increased (Martin et al., 1996). This interpretation has also been supported by connectionist implementations of the decay hypothesis within an interactive spreading activation model (Martin et al., 1996; Foygel and Dell, 2000). In sum, these patients, provide compelling evidence for a STM-based repetition impairment, and given their lesion overlap in the left posterior temporo/parietal area, can be considered to show impairment to the pSTG hub region of the dorsal language repetition stream.

A further argument often invoked for localizing phonological maintenance processes in the pSTG/inferior parietal area is the documentation of patients with specific phonological STM deficits: these patients typically show relatively spared single word repetition, but a severe reduction of multi-word repetition abilities, in association with lesions in the posterior superior temporal area extending to the supramarginal gyrus and the arcuate fasciculus, i.e., the dorsal repetition pathway (e.g., Warrington et al., 1971; Vallar et al., 1990; Basso et al., 1982; Majerus et al., 2004a; Takayama et al., 2004). This was also supported by early neuroimaging studies of verbal short-term maintenance, locating the verbal short-term storage function to the same posterior temporo-parietal neural substrate (Paulesu et al., 1993; Salmon et al., 1996; see Becker et al., 1999; Chein and Fiez, 2001 for an exhaustive review of these studies). These data suggest a close association between STM deficits in the phonological domain and lesions in the posterior part of the dorsal repetition pathway.

A different type of patients has been described with difficulties in maintaining semantic information during language repetition and comprehension tasks (e.g., Martin and Romani, 1994; Martin et al., 1994b, 1999; Freedman and Martin, 2001; Martin and He, 2004; Hoffman et al., 2009; Barde et al., 2010). The lesion involved here is located in the more anterior part of the left inferior prefrontal cortex and/or middle and inferior temporal cortex, which is part of the ventral stream of language processing (Hickok and Poeppel, 2007; Friederici, 2012), suggesting that the ventral pathway is also related to maintenance aspects during language reproduction, and this more specifically for semantic information [R. (Martin et al., 1994b, 1999); however, see Barde et al. (2010) for further involvement of the left angular gyrus area in some patients]. More specifically, these patients are generally poor in maintaining semantic information during sentence repetition/comprehension and show diminished lexicality and semantic effects during language repetition, as well as an increased rate of intrusion errors (Martin et al., 1994b, 1999). Although initially attributed to a semantic buffer deficit, their semantic maintenance difficulties have subsequently been linked to difficulties in inhibiting previously activated items, and have recently been related to a more general semantic control deficit^1^ (Hamilton and Martin, 2005, 2007; Jefferies et al., 2007; Hoffman et al., 2009). A second type of intervention of the ventral language pathway in maintaining semantic information in STM is illustrated by patients showing loss of semantic information, as is the case in patients with semantic dementia (Hodges et al., 1992). These patients present a progressive loss of semantic representations, with lesions typically involving the ventral speech stream, gray matter loss starting in inferior, anterior and medial regions of the temporal lobe and involving also the anterior inferior prefrontal and orbito-frontal cortex (Mummery et al., 2000; Good et al., 2002; Desgranges et al., 2007). During language repetition, in both single and multiple word/nonword repetition tasks, patients with semantic dementia present a marked reduction of lexicality effects, with word spans being severely impaired but non-word spans often remaining in the normal range (Patterson et al., 1994; Knott et al., 1997; Majerus et al., 2007a). The data from patients with semantic dementia show that temporary activation of long-term memory lexico-semantic representations is a further critical determinant of language repetition and maintenance. In sum, the data from patients with selective semantic STM or semantic knowledge impairment suggest that the ventral repetition pathway is involved in language maintenance processes by providing the necessary substrate for activation and representation the semantic information to be maintained, and by supporting semantic control processes which protect semantic memoranda against semantic intrusions.

A straightforward conclusion of these results, and which is a more or less implicit assumption of recent language processing models and language-based STM models (Martin and Saffran, 1992; Hickok and Poeppel, 2007; Acheson and MacDonald, 2009; Friederici, 2012; Hickok, 2012), is that temporary maintenance of phonological information during language repetition depends upon the dorsal pathway, and that temporary maintenance of semantic information during language repetition depends on the ventral pathway. In other words, the language processing networks could be considered to be sufficient for supporting short-term maintenance in language repetition tasks, via temporary activation of phonological, sensori-motor interface and semantic representations, and this from the encoding stage until the response is produced. Verbal information is considered here to be maintained via continuous activation all over the maintenance phase of underlying language representations initially activated during encoding (Martin and Saffran, 1992). This conclusion, although parsimonious, however, does not take into account the results of studies that have more directly explored the neural substrates of verbal short-term maintenance. In the STM research field, load effects are considered to be a core characteristic of maintenance processes: the higher the number of stimuli to be maintained, the higher the maintenance load, and the greater the solicitation of maintenance processes. This implies that regions involved in temporary maintenance of verbal information should be sensitive to maintenance load (Postle, 2006). Neural substrates supporting load effects have indeed been identified, but these typically involve areas outside dorsal and ventral repetition pathways: the superior parietal and intraparietal cortex as well as the dorsolateral prefrontal cortex have been shown to be sensitive to STM load (Awh et al., 1999; Rypma and D'Esposito, 1999; Leung et al., 2002, 2004; Rypma et al., 2002; Ranganath et al., 2004; Ravizza et al., 2004; Narayanan et al., 2005; Majerus et al., 2012). Similarly, Martin et al. (2003) investigated phonological and semantic maintenance processes by exploring load effects in phonological (rhyme judgment) and semantic (category judgment) STM tasks: for both tasks, load effects were observed outside the ventral and dorsal language processing pathways, and involved the superior parietal cortex, the intraparietal sulcus and the dorsolateral prefrontal cortex in the left hemisphere; activations in the language network were observed, with specific recruitment of regions of the dorsal language pathway (left supramarginal cortex) for the rhyme judgment task but these activations did not respond in a load-dependent manner. These results suggest that while dorsal and ventral language pathways are specialized in representing phonological and semantic information, respectively, they do not reflect maintenance processes per se if we consider these to be defined by load effects.

These data should, however, not be taken as a conclusive argument against a role of temporary activation of language representations during STM maintenance. Studies will need to assess load-effects in dorsal and ventral pathways using more sensitive neuroimaging methods such as multivariate voxel pattern analyses; these techniques have recently allowed to show load effects in occipital visual processing areas during visual maintenance tasks where univariate analyses failed to reveal any such effects (Emrich et al., 2013). Maintenance of six vs. two verbal items may not necessarily be associated with differential activation levels in language processing areas, as measured with traditional univariate analyses, but could rely on more subtle differences in activation patterns. Enhanced activation levels in fronto-parietal cortex areas may on the other hand reflect increasing attentional demands for distinguishing increasingly overlapping activation patterns in the language processing areas when maintaining six vs. two items. Also, most patients with apparent selective phonological STM impairment are likely to have difficulties at the level of processing and maintaining language representations. These patients typically present lesions in the dorsal language network (posterior superior temporal and temporo-parieral cortex) rather than the load-dependent fronto-parietal networks (Warrington et al., 1971; Basso et al., 1982; Vallar et al., 1990; Majerus et al., 2004a; Takayama et al., 2004). The vast majority of these patients show a history of aphasia, with residual phonological processing deficits in most cases (see Majerus, 2009). In their meta-analysis, Majerus (2009) showed a strong positive correlation between the severity of the phonological STM impairment and residual language processing impairment, suggesting that residual difficulties in representing and processing phonological information may at least partially explain phonological STM deficits in these patients (see also Buchsbaum and D'Esposito, 2008 and Postle, 2006).

Overall, the results from the language processing and STM research fields appear to be conflicting. On the one hand, the neuropsychological data reviewed here suggest that impairment at the dorsal and ventral language pathways is clearly associated with difficulties in tasks involving the maintenance of phonological and semantic information, respectively. On the other hand, studies from the STM research field highlight bilateral fronto-parietal networks as being related to core STM processes such as load effects. In order to understand this apparent paradox, we need to examine more deeply the nature of representations and processes involved in the maintenance of verbal information. In order to achieve this, a three-component framework is proposed here. In this architecture, a first component is related to temporary activation of language representations in the dorsal and ventral language processing networks: to-be-repeated language stimuli have to be represented at the item level that is, their phonological and lexico-semantic features need to be encoded, represented and maintained, and this will be achieved via continuous activation in the language pathways. Second, the serial order of the stimuli needs to be represented: this is more particularly the case when sequence information of the words or phonemes within the string of memoranda is unfamiliar. Third, although language repetition is a simple, straightforward task and will not require demanding executive control processes, at least in healthy, language non-impaired individuals, attentional focalization on the target stimulus/stimuli will be required ad minima, and these requirements will increase with increasing number and decreasing familiarity of the verbal stimuli to be repeated. The latter two components are proposed to be supported by the load-dependent, fronto-parietal networks typically associated with STM tasks. In the following sections, we will discuss empirical support for this three-component architecture of temporary maintenance for verbal stimuli.

## Language repetition pathways and maintenance of item information

Dorsal and ventral language processing networks are proposed here to have one specific function during verbal maintenance: they provide the representational basis for the encoding of phonological and semantic features of the items to be maintained and repeated. In other words, via its temporary activation, the language network ensures the encoding and representation of phonological and semantic item information during temporary maintenance of verbal information. Critically, this excludes the representation of novel serial order information, such as the arbitrary ordering of the words within a list of words to be repeated, such as a phone number. This distinction between the representation of item information and serial order information is in short-term maintenance tasks defines most of recent verbal STM models and is supported by empirical evidence that will be presented in this and the next section. These STM models consider that during maintenance of verbal information, verbal item information is directly represented within the language system, rather than by a copy in a dedicated STM buffer, while the representation of novel serial order will be processed via a specific serial order processing system to which the language system is connected (Burgess and Hitch, 1999, 2006; Martin et al., 1999; Brown et al., 2000; Gupta, 2003). Verbal item information is considered to be maintained via sustained activation of the phonological representations and semantic representations along the dorsal and ventral repetition pathways and which have also served to process the target item during perception and encoding.

The assumption that language processing networks mainly serve to represent phonological and semantic item information is supported by a number of behavioral and neuroimaging studies. At the behavioral level, it is well-established that linguistic variables, such as word frequency, word imageability and semantic valence and richness will determine the amount of item information that is correctly recalled in a word list immediate serial recall task (i.e., the number of items independently of their serial position), but not recall of serial order information (i.e., the number of items within correct serial position) (Hulme et al., 1991; Poirier and Saint-Aubin, 1996; Nairne and Kelley, 2004; Majerus and D'Argembeau, 2011). This shows that access to linguistic levels of representation affects maintenance of verbal item information, but not within-list serial position information. Second, neuroimaging studies have shown that, when maintaining items and their phonological characteristics, phonological processing areas in the pSTG area and adjacent inferior parietal cortex are activated at least during the initial stages of maintenance (Collette et al., 2001; Martin et al., 2003; Majerus et al., 2006a, 2010; Pa et al., 2008; Gettigan et al., 2011). Furthermore, in a recent MEG study, Herman et al. (2013) showed that processing of long non-word sequences, involving a delay below stimulus input and repetition, were associated with increased reverberating activity between posterior (temporo-parietal) and anterior (inferior frontal) sites of the dorsal pathway, suggesting sustained and synchronized activation of input and output phonological representations during maintenance of verbal stimuli. Similarly, when maintaining semantic item information, semantic processing areas in the inferior temporal lobe have been shown to present sustained activation over the maintenance interval (Fiebach et al., 2006, 2007). These data suggest that ventral and dorsal language pathways are involved in maintenance during language reproduction tasks, by providing the representational substrates necessary for encoding and representing the items, i.e., the phonological and semantic characteristics of the information to be maintained and repeated.

This is further supported by patients with semantic processing deficits. These patients typically present difficulties in repeating semantic item information, but not serial order information, with serial order recall being perfectly preserved (Majerus et al., 2007a; Papagno et al., 2013). These patients produce very specific item error patterns in repetition tasks, the so-called blending errors where phonological forms of different words are recombined to form nonsense phonological forms. Although these errors could be considered to reflect syllable or phoneme ordering errors, they are in fact a direct consequence of the loss of semantic information: lexico-semantic knowledge normally binds the phonological segments defining a word form to its semantic referent allowing for robust phonological item representations; if this knowledge is degraded, lexical phonological representations for a given word degrade, the word being processed like a non-word and leading to the phonological recombination errors which are typically observed when healthy participants repeat sequences of non-words (Treiman and Danis, 1988; Patterson et al., 1994; Jefferies et al., 2005; Acheson and MacDonald, 2009). In support of this interpretation, Jefferies et al. (2006) have shown that healthy adults conduct the same type of phonological recombination errors in word list immediate serial recall tasks when word stimuli are not recognized as lexical items anymore, for example when presented together with non-words in mixed and unpredictable word-nonword list repetition designs. More generally, these data also show that in the absence of long-term language knowledge, serial order information of phoneme order is difficult to maintain and serial ordering errors appear during repetition performance.

Finally, syntactic information will also support maintenance and recall of information, by binding item information and item order via long-term syntactic structures. This will again be the case for familiar information such as coherent sentences with canonical sentence structure. A number of studies have shown that verbal STM span can be significantly increased when presenting word lists organized as sentences; in this case word span will increase to about 16 words (Brener, 1940; Baddeley et al., 1984, 2009). Although this finding has been attributed to increased opportunities for the use of chunking processes, a straightforward interpretation of these results is the intervention of syntactic and conceptual long-term memory structures which will determine the syntactic and conceptual relations between the items, and therefore also their position in a sentence structure (Garrett, 1980). For example, the pronoun “the” will always precede its corresponding noun, and the sentence subject will precede the verb while the object will follow the verb for canonical sentence structures. At the conceptual level, the agent will generally precede the action and the beneficiary. This knowledge, embedded in the ventral pathway for the conceptual aspects and in the dorsal pathway for the syntactic aspects, will support both item and order recall in a sentence context (Friederici, 2012). However, if incoherent sentences are presented with words in scrambled order, sentence span will decrease and especially serial order errors will appear (Hoffman et al., 2012). In other words, the representations of the language system are able to support familiar item and order information, but not unfamiliar order information, as has already been shown for non-word repetition.

In sum, these data provide support for language representations in the dorsal and ventral speech streams as providing the representational basis for temporary maintenance of *item* information. Language processing models, such as those developed by Hickok and Poeppel (2007), Jacquemot and Scott (2006) and Friederici (2012), and recent STM models mentioned in this section show strong theoretical convergence here, both considering that temporary activation of long-term representations in the language network is a critical step of verbal maintenance. However, temporary activation of representations in the dorsal and ventral language bases are not the only processes that intervene during short-term maintenance of verbal information, and it is at this point that language processing and STM models start to diverge.

## The role of fronto-parietal networks in verbal maintenance: serial order processing

A hallmark characteristic of many recent verbal STM models is the consideration of mechanisms that allow for the temporary maintenance and reproduction of arbitrary sequence information that is, the ability to recall verbal items/phonemes as a function of their serial position during list/stimulus presentation (Henson, 1998; Burgess and Hitch, 1999, 2006; Brown et al., 2000; Gupta, 2003; Botvinick and Watanabe, 2007). Language processing models rarely consider this ability but assume that serial order information is an inherent part of linguistic structure and is supported by linguistic structure during language reproduction (Acheson and MacDonald, 2009; see also Postle, 2006). This assumption is valid when linguistic structure knowledge is available. The ordering of phonemes for familiar word forms will be determined by phoneme- and syllable-cooccurrence and transition probabilities encoded in phonological representations; the same will also be true for non-words, where sublexical phonotactic knowledge and syllable structure knowledge will determine output in non-word recall tasks (Treiman and Danis, 1988; Vitevitch and Luce, 1999; Dell et al., 2000; Majerus et al., 2004b; Acheson and MacDonald, 2009; Gupta and Tisdale, 2009). Likewise, during sentence repetition, syntactic and conceptual knowledge will constrain the order of the words during output (Dell, 1986). However, when this knowledge is not available serial order errors will occur during repetition. This is illustrated by non-word recall where sublexical phonotactic knowledge is not sufficient to accurately encode and reproduce the serial ordering of the phonemes, especially if the underlying phonological pattern of the non-word is highly unfamiliar; in that case, errors during non-word repetition will start to appear and these errors will be mainly phoneme order errors (Treiman and Danis, 1988; Gupta et al., 2005; Jefferies et al., 2006). This is also the case when the order of an arbitrary list of words needs to be maintained and repeated, such as when repeating a phone number, a list of unrelated words, a novel sequence of task instructions or a novel sequence of orally given directions.

While in the STM domain, many detailed models of the processes supporting the maintenance and reproduction of novel sequence information have been developed (e.g., Henson, 1998; Burgess and Hitch, 1999, 2006; Brown et al., 2000; Gupta, 2003; Botvinick and Plaut, 2006), the neural pathways associated with these processes have only been recently uncovered. Studies exploring the neural substrates associated with serial order maintenance and reproduction have observed a critical role of the inferior parietal cortex, and more specifically the intraparietal sulcus area. Marshuetz et al. (2000) observed higher activation in bilateral intraparietal sulci when maintaining the serial order of arbitrary letter sequences as opposed to maintaining letter identity (see also Marshuetz et al., 2006). When comparing serial order and item STM conditions with a stricter control of task difficulty, Majerus et al. (2006a, 2010) observed that maintenance and retrieval of serial order information for word lists as well as non-word lists is restricted to activation in the right intraparietal sulcus, in addition to activation in the bilateral superior frontal cortex and the right superior cerebellum; the superior frontal cortex contribution to serial order processing has also been observed by Henson et al. (2000) and has been associated with serial regrouping. On the other hand, activation is stronger in the dorsal and ventral language networks when maintaining item identity information such as in conditions where participants have to focus on and later recognize the phonological, orthographic or semantic characteristics of the memoranda (Majerus et al., 2006a). Furthermore, the fronto-parietal network supporting encoding and maintenance of serial order information appears to be domain general, the same network having been shown to be also involved in the short-term maintenance of serial order information for visual sequences such as sequences of unfamiliar faces (Majerus et al., 2007b, 2010).

The separation between language -based item maintenance processes, and serial order maintenance processes is also confirmed by patients presenting verbal STM deficits. Case studies with double dissociations between item-based and order-based maintenance deficits have been documented. Attout et al. (2012) described two patients, MB and CG, with poor performance in verbal repetition and reproduction tasks and poor digit spans. An exhaustive exploration of MB's performance profile for STM tasks maximizing either the retention of verbal item information or serial order information showed that patient MB had difficulties mainly in recognizing and reproducing item information; word and non-word list repetition was characterized by a significantly increased rate of omissions errors and phonological paraphasias but his serial recall was perfect: all words correctly recalled were reproduced in correct serial position. His item-based STM impairment was furthermore associated with a mild residual phonological processing impairment, in the context of a left posterior peri-sylvian cerebro-vascular accident. On the other hand, CG, a patient with traumatic brain injury^2^, showed the reverse profile: he showed an abnormally high rate of serial ordering errors in verbal repetition tasks, while showing perfect item reproduction abilities; he recalled as many items as controls, but had substantial difficulties in outputting the items in correct serial position. The existence of a double dissociation between item and order verbal maintenance deficits is also an important argument against unitary models of verbal STM such as the model by Botvinick and Plaut (2006) considering that item and order information are bound in a single representation during maintenance and reproduction of verbal sequential information. These dissociations are also contradicting language-based serial order coding accounts, where the maintenance of serial order is supposed to be achieved mainly via repeated cycling of the input sequence through the language production system (Page and Norris, 1998; Postle, 2006; Page et al., 2007; Acheson and MacDonald, 2009). As already noted, phonological, semantic and syntactic linguistic structures will support serial recall if the sequence information can be mapped onto existing long-term memory sequence structures (such as syllable frames, phonotactic constraints, lexical word form representations, scripts), however, this will not be possible when the sequence information is novel, arbitrary and highly unfamiliar (Treiman and Danis, 1988; Gupta et al., 2005; Jefferies et al., 2006). The evidence presented here is in favor of separate cognitive and neural substrates supporting item vs. order representation in language maintenance and reproduction tasks.

Importantly, the distinction between item and order maintenance capacities has further functional implications for language processing. The serial order maintenance capacities supporting novel sequence reproduction may be critical for language repetition and learning. A number of behavioral studies have shown that repetition and learning of novel phonological sequences is most strongly associated with serial order maintenance capacities, as opposed to item maintenance capacities: children and adults showing high serial order maintenance capacities as measured by serial order reproduction and reconstruction tasks have larger vocabulary knowledge bases and learn faster novel vocabulary; item STM tasks involving item recall independently of serial order position information are more weakly associated with performance in novel word repetition and learning tasks (Majerus et al., 2006b,c, 2008a; Mosse and Jarrold, 2008; Leclercq and Majerus, 2010). A theoretical interpretation of these findings is that the ability to temporarily maintain sequence information via a dedicated short-term storage system for order information allows the unfamiliar phoneme sequences which define a novel word to be maintained and replayed in correct order during the repetition and learning process, thereby increasing the strength of the new lexical phonological representation being created in the language knowledge base; this entails that the language pathways (where item representations—phonemes/syllables/complete word forms—are stored, temporarily activated and learnt) and the order maintenance system are interconnected and in close interaction (Gupta and MacWhinney, 1997; Burgess and Hitch, 1999, 2006; Gupta, 2003).

In the light of these data, we should expect that, at the neural level, single novel word repetition and learning is also associated with the fronto-parietal serial order processing network. In support of this hypothesis, Majerus et al. (2008b) observed a correlation between novel word learning capacities in healthy adults and the recruitment of the frontal part of the fronto-parietal serial order processing network. In the same vein, a MEG study exploring the time course of brain activity associated with the repetition of non-word syllable sequences, observed, in parallel to reverberating activity in the dorsal language pathway, an involvement of the right intraparietal sulcus area; the non-word syllable repetition task used in that study had strong serial order processing requirements, since the different non-word sequences were sampled each time from the same set of three syllables (ba, da, or pa) with syllable serial order being the distinguishing feature between the different non-word sequences (Herman et al., 2013); furthermore, the right IPS involvement was not just coincidental, but it was associated with behavioral success during the syllable repetition task. On the other hand, other studies investigating the neural substrates of novel word repetition or maintenance have observed activation restricted mainly to the dorsal language pathway (e.g., Strand et al., 2008; Papoutsi et al., 2009; Gettigan et al., 2011). These studies, however, most often control for task-general factors by including baseline conditions which factor out neural activation related to serial order processing, such as via the use of tone sequence processing conditions (Strand et al., 2008; Gettigan et al., 2011). As we have noted, the fronto-parietal network supporting serial order processing for verbal tasks also supports ordinal processing in other modalities (Majerus et al., 2007b, 2010; Dormal et al., 2012). By using these control conditions, the intervention of fronto-parietal serial processing mechanisms may have been masked. Similarly, other studies contrasted non-word conditions that varied according to a number of linguistic dimensions (such as articulatory constraints) and where the reference non-word condition already included serial order processing, which may again have masked the potential intervention of fronto- parietal serial order processes (Papoutsi et al., 2009). In sum, the intervention of fronto-parietal serial order processing mechanisms has been established for the maintenance and reproduction of order information in word, non-word and letter sequences; the conditions under which these processes also intervene in single non-word processing have to be further investigated.

## The role of frontoparietal networks in verbal maintenance: attentional focalization

A second hallmark feature of recent verbal STM models, and which is considered even less by language architectures than serial order processing, is attentional processing. Many recent models of STM consider that maintenance of verbal information does not only require temporary activation of language representations, but the maintenance of this activation over time until task completion is further under the control of attentional focalization processes (Cowan, 1995; Oberauer, 2002; Barrouillet et al., 2004; Engle and Kane, 2004). Although the role of attention has been acknowledged by early STM models, such as the working memory model by Baddeley and Hitch (1974), recent data show that attentional focalization intervenes not only in complex storage and processing tasks, but also in simple verbal tasks requiring only maintenance and output of a set of stimuli as is the case of language repetition tasks (Cowan et al., 2005; Majerus et al., 2009; Ötzekin et al., 2010). In other words, temporarily activated representations are considered to remain activated as long as required by being put in the focus of attention and by being re-activated each time they are the target of the focus of attention (Cowan, 1988, 1995). Direct neuroimaging evidence for this mechanism has been observed in the area of face processing, where Gratton et al. (2013) recently showed that items hold in the focus of attention are characterized by enhanced neural response in temporo-occipital face processing areas relative to items outside the focus of attention. This control of activation maintenance via attentional processes will further allow to ensure that activated input and output representations match, possibly via additional efference copies sent to the inferior parietal cortex, allowing that input information is correctly reproduced at output (Rauschecker and Scott, 2009). Attentional capacity is currently considered by many authors to be the core limiting factor of performance in verbal maintenance tasks, and the defining factor of maintenance capacity (Cowan, 1995; Oberauer, 2002; Barrouillet et al., 2004; Engle and Kane, 2004).

At the neuroimaging level, part of the fronto-parietal network that typically defines the neural substrates of verbal maintenance tasks has been associated with this attentional focalization function, and this more precisely at the level of the left intraparietal sulcus and the dorso-lateral prefrontal cortex (Salmon et al., 1996; Nystrom et al., 2000; Ravizza et al., 2004; Cowan et al., 2011; Majerus et al., 2012). Although bilateral fronto-parietal activity is typically observed in verbal maintenance tasks, only the left intraparietal sulcus and dorsolateral prefrontal cortex appears to be activated irrespective of the type of information to be maintained, and is considered to have a domain-general attentional control and focalization function in STM tasks (Majerus et al., 2010; Cowan et al., 2011). The right intraparietal sulcus area appears to have a more specific function and is activated more strongly when maintaining serial order information as we have seen in the previous section. This fronto-parietal network is also considered to support attentional focalization processes rather than a verbal buffer function since this network is sensitive to load-effects not only in the verbal domain, but also when temporarily maintaining other types of information such as faces, geometric stimuli, tactile stimuli or even social stimuli (e.g., Nystrom et al., 2000; Rämä et al., 2001; Hautzel et al., 2002; Todd and Marois, 2004; Brahmbhatt et al., 2008; Lycke et al., 2008; Meyer et al., 2012; Kaas et al., 2013). This has led to the currently dominant view in the STM research field that an important function of the fronto-parietal network is the control of task-related attention during the maintenance of verbal information, allowing attention to be directed and maintained on the target stimuli to be processed and maintained (Todd and Marois, 2004; Postle, 2006; Nee and Jonides, 2011, 2013). More specifically, this STM load-dependent fronto-parietal network has been shown to involve a well-known network in the attention research field, the dorsal attention network which allows attention to be oriented on target stimuli as a function of ongoing task requirements, in both verbal and visual domains (Todd and Marois, 2004; Majerus et al., 2012). This network has been shown to increase its activity with increasing STM load, while competing with a second attentional network during verbal maintenance tasks, the ventral attention network involved in detecting novel, task-irrelevant stimuli; the ventral network, involving the temporo-parietal junction and the orbito-frontal cortex, is deactivated as a function of the amount of verbal stimuli to be maintained, and this deactivation is associated with attentional blindness for distractor stimuli presented while the verbal stimuli are being maintained (Todd et al., 2005; Fougnie and Marois, 2007; Majerus et al., 2012). These data demonstrate the central role of task-related attentional processes as defining left-hemisphere fronto-parietal activity during maintenance of verbal stimuli.

In the light of these data, any model representing language repetition and maintenance processes should consider interactions with these domain-general attention networks, since they have been shown to be one of the main function of the left-centered fronto-parietal network recruited during temporary maintenance of verbal information. Furthermore, repetition of multiple word or non-word sequences will particularly require attentional control processes in order to ensure that input and output match (Rauschecker and Scott, 2009). While this network is consistently observed to be involved in tasks involving the maintenance and reproduction of multiple word or non-word stimuli (Ravizza et al., 2004; Majerus et al., 2006a, 2010; Ötzekin et al., 2010; Cowan et al., 2011), this is less consistently the case for single word and non-word repetition. On the one hand, single word repetition will probably require attentional focalization processes only to a minimal amount, since a single target has to be processed and the maintenance delay is very short due to a quasi-immediate succession of input and output processes. This is also in line with neuroimaging studies of verbal maintenance showing no or minimal recruitment of left fronto-parietal networks in low-load conditions (e.g., when a single or two letters have to be maintained; Majerus et al., 2012). However, this may be different for single non-word repetition, especially if the phonological structure of the non-word is highly unfamiliar and multisyllabic and will be difficult to map onto existing sublexical phonological representations along the dorsal language pathway. In that case, fronto-parietal maintenance mechanisms are likely to be challenged to a higher extent. Studies having investigated the neural substrates of single non-word repetition do not systematically observe activation of the left-centered fronto-parietal network (Pa et al., 2008; Strand et al., 2008; Papoutsi et al., 2009; Gettigan et al., 2011). This could, however, be related to the control conditions used in these studies, factoring out domain-general processes such as attentional focalization. As already noted in the previous section, most of these studies aim at exploring neural activations specifically associated with linguistic processing and maintenance, and therefore use baseline conditions which remove more general cognitive variables, for example by presenting tone or gesture sequences to be processed and maintained as a reference condition (e.g., Pa et al., 2008; Gettigan et al., 2011). On the other hand, when considering activations shared with processing of the control conditions, activation in intraparietal areas can be observed, as was for example the case in the study by Pa et al. (2008) comparing speech and gesture maintenance. Also, in their recent MEG study exploring the time course of neural activation during language repetition, Herman et al. (2013) observed activation in the left fronto-parietal network during non-word repetition. Interestingly, this network reacted in a load-dependent manner, with higher recruitment for repetition of 4-syllable non-words as compared to two-syllable non-words. Furthermore, it is important to note here that the involvement of the left fronto-parietal network occurred at a relatively late time point after encoding, between 500 and 700 ms post-stimulus onset, while activation in the dorsal language network was present about 40 ms post-stimulus onset. This later involvement of the fronto-parietal network is in line with its top-down attentional control function during language processing: language representations of to-be-repeated stimuli are first activated in the language processing networks, and their activation is then maintained and monitored via top-down task-related attentional control. Finally, in a recent neuroimaging study exploring functional connectivity patterns during a sentence processing task, Makuuchi and Friederici (2013) found further evidence for the involvement of fronto-parietal networks in language processing tasks. Using dynamic causal modeling, they observed functional connectivity between the left-hemisphere fronto-parietal network and core language processing areas during sentence processing, and the strength of this association increased as a function of the linguistic complexity of the verbal material and, by extension, of the amount of attentional focalization/control needed. These data show that the fronto-parietal network is not only co-activated during language processing tasks, but is an integral and integrated part of language processing networks.

## Toward an integrative framework for maintenance processes during language repetition

In this review, aiming at elucidating the cognitive processes and neural networks involved in the maintenance of verbal information during language repetition, we have shown that research in the language and STM dosmains converge on one important factor: the importance of language knowledge supported by the dorsal and ventral pathways and its temporary activation during maintenance of verbal information. On the other hand, research in the STM domain points to two additional processes: those involved in maintaining novel sequence information, and those involved in maintenance control via attentional focalization processes. Although language processing models have given no or very little consideration to the latter two processes, the studies reviewed here show that temporary maintenance of verbal information can depend on all three factors identified here, especially when multiple word stimuli or long non-word stimuli need to be processed. Similarly, cognitive architectures of STM consider interactions between either language processing and serial order processing (e.g., Brown et al., 2000; Gupta, 2003; Burgess and Hitch, 2006), or language processing and attentional processing (e.g., Cowan, 1995; Oberauer, 2002; Barrouillet et al., 2004), but no STM model currently considers the three components of verbal maintenance identified here at the same time.

**Figure 1:**
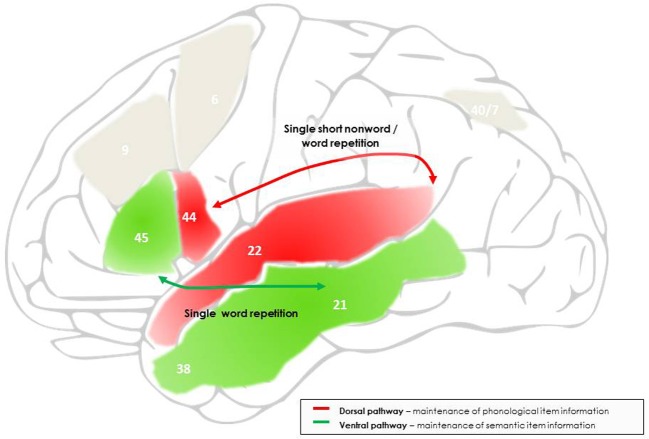
**Outline of the networks and processes proposed to support maintenance processes during single word and short non-word repetition**. Maintenance during short non-word repetition is mainly supported by the dorsal language pathway, linking the superior and posterior temporal cortex to the posterior inferior frontal cortex, and, at the cognitive level, reflects temporary activation and interfacing of input and output phonological item representations. Maintenance of single word repetition is also supported by the dorsal language pathway, but with additional intervention of the ventral language pathway, linking the middle and anterior temporal cortex to a more anterior site of the inferior frontal cortex, and reflects temporary activation of semantic item representations. The frontal endpoints of each pathway are further involved in protecting to-be-maintained information against phonological and semantic interference, respectively. The numbers indicate the main Brodman areas characterizing each functional region identified here.

**Figure 2:**
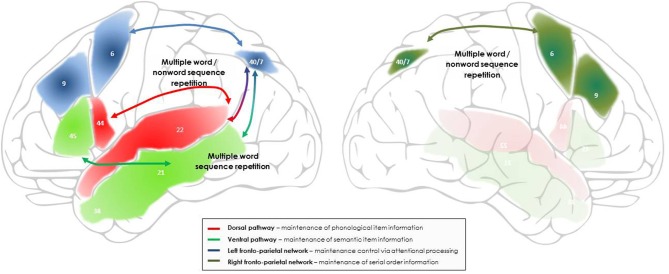
**Outline of the networks and processes proposed to support maintenance during multiple non-word and word sequence repetition, including sentence repetition**. Bilateral fronto-parietal networks, supporting domain-general attentional and serial order processing, intervene in addition to the dorsal and ventral language pathways involved in the maintenance of phonological and semantic item information. In the left hemisphere, the fronto-parietal network associating the left intraparietal sulcus to the left superior and middle prefrontal cortex is proposed to support the maintenance of multiple verbal stimuli by focusing attentional resources on the representations temporarily activated in the dorsal and ventral language pathways. In the right hemisphere, the fronto-parietal network associating the right intraparietal sulcus to the right superior and middle prefrontal cortex is proposed to support maintenance of the novel and unfamiliar serial order information that characterizes the order of occurrence of words within a list and of phonemes/syllables within a novel word, via connectivity with the left fronto-parietal network and the language pathways. The numbers indicate the main Brodman areas characterizing each functional region identified here.

The integrative framework of verbal maintenance processes during language repetition proposed here considers language, serial order and attention components within a single model. An overview of this functional architecture and underlying neural networks is presented in Figure 1, for single word and non-word repetition, and in Figure 2, for word and non-word sequence repetition including sentence repetition. The basis of this architecture are the dorsal and ventral language pathways, where long-term phonological and semantic representations are activated upon presentation of a word (see Figure 1). More precisely, in the dorsal network, sublexical phonological representations in the posterior superior temporal area and the superior temporal sulcus will be activated and temporarily maintained (Binder et al., 2000; Scott et al., 2000); two different types of representation may be distinguished here: the posterior superior temporal area (planum temporale) has been proposed to support sensori-motor interface representations, which, in direct connection with the inferior frontal cortex, will allow target representations to get continuously reactivated and refreshed via subvocal articulatory rehearsal processes (the “doing” pathway; Hickok and Poeppel, 2007; Rauschecker and Scott, 2009); the more anterior superior temporal areas and superior temporal sulcus have been proposed to keep track of the initial perceptual properties of the target information (Buchsbaum et al., 2005). In the ventral network, activations in the anterior, middle and inferior temporal areas will represent the lexical and semantic properties of the target information (Scott et al., 2000; Binder et al., 2000, 2009; Friederici, 2012). Importantly, at this stage only item representations will be activated and maintained, allowing individual words to be maintained and repeated on the basis of their underlying phonological, lexical and semantic representations. However, as we have seen, these representations will not be sufficient to maintain sequence information, i.e., to maintain the (arbitrary) serial order in which the different words have been presented. Activation in the language pathways therefore needs to be synchronized with an additional system which allows for the coding of arbitrary sequence information (see Figure 2): this function is proposed to be supported by a fronto-parietal network centered on the right intraparietal sulcus, which will associate each activated item in the language network with a serial position marker ensuring that each item will be output in correct serial position at recall, as proposed by a number of computational models of serial order STM (Gupta and MacWhinney, 1997; Burgess and Hitch, 1999, 2006; Brown et al., 2000). Finally, attentional control will be needed to maintain the item and serial order representations activated over time and in the focus of attention, as a function of current task requirements. This function is proposed here to be supported by a fronto-parietal network centered around the left intra-parietal sulcus (Figure 2), in line with an increasing number of studies associating the fronto-parietal activations during verbal and non-verbal maintenance with the dorsal attention network (Todd and Marois, 2004; Cowan et al., 2011; Majerus et al., 2012). This network will interact with the other two networks in order to ensure synchronized activation and processing, which will lead to successful task performance and accurate reproduction of both item and order information.

This architecture of verbal maintenance is considered to be task-dependent: when repeating single words or short non-words with a familiar sublexical phonological structure in an immediate repetition task, processing is likely to be limited to the ventral and dorsal repetition pathways, respectively, since there will be no novel serial position information to be processed; requirements for extended maintenance via attentional focalization will also be minimal since the perceptual input will be processed by the language repetition pathways in a quasi instantaneous manner and the target item activation does not need to be protected against competitor stimuli. In accordance, patients with a severe single word repetition impairment often have lesions restricted to these pathways, and more precisely, the posterior part of the dorsal pathway (Buchsbaum et al., 2011). Furthermore, recent studies exploring the role of attentional processes on maintenance processes have shown that at output, not all information will be in the focus of attention, and some information can be directly retrieved from activated long-term memory (Nee and Jonides, 2008, 2011, 2013; Lewis-Peacock et al., 2012). Similarly, when repeating multiple word sequences, the serial order processing network may not be extensively recruited if output in correct serial position is not required. Previous studies have shown that healthy subjects can recruit the serial order processing network centered around the right intraparietal sulcus as a function of task demands: when processing of sequential aspects is stressed by task instructions, stronger recruitment of the right intraparietal sulcus is observed; but when task instructions focus on the maintenance of phonological and orthographic characteristics of the items, dorsal and ventral language processing streams are activated more strongly (Majerus et al., 2006a, 2010). The flexible recruitment of these different networks is supposed to be under the control of the fronto-parietal network centered around the left intraparietal sulcus involved in top-down attentional processing. For tasks with varying item and serial order processing demands, the left intraparietal sulcus has indeed been shown to be activated for both types of information but with differential functional connectivity patterns, connectivity being enhanced between the left intraparietal sulcus and language processing networks when item processing demands are high, and connectivity being enhanced between the left and right intraparietal sulci when serial processing demands are high (Majerus et al., 2006a, 2008b). These data suggest that attentional control by the left fronto-parietal network can be flexibly allocated to language processing and/or serial order processing networks, as a function of task demands.

A number of predictions are to be derived from the framework proposed here. First, a strong prediction of this framework is the greater involvement of the serial order processing and attentional processing components during non-word repetition, especially when the non-word sequence is long, complex and cannot be easily mapped to existing lexical and sublexical phonological structures, i.e., non-words with very low lexical neighborhood values and phonotactic probability values. In that case, the sequence of phonemes cannot be represented via existing sublexical phonological structures, and the novel sequence information needs to be maintained via strong connections between the phonological item representations supported by the dorsal repetition pathway, the novel sequence representations supported by the fronto-parietal network centered around the right intraparietal sulcus, and attentional resources supported by the fronto-parietal network centered around the left intraparietal sulcus (see Figure 2). As already mentioned, previous studies exploring the neural substrates of non-word repetition typically focused on the linguistic networks and/or used control conditions factoring out any possible contribution of the serial order and attention processing components identified here (e.g., Gettigan et al., 2011). In support of this, studies looking directly at the time course of activation patterns during non-word repetition, without using any baseline condition, observed in addition to involvement of the dorsal language pathway activation in left and right inferior parietal areas which was stronger for longer non-word sequences. Moreover, if output is delayed, there will be additional requirements for short-term maintenance, and in that case, the intervention of attention networks may be necessary for maintaining active the corresponding phonological representations even for short non-words. Future studies will need to determine in a systematic manner the conditions in which serial order and attention processing networks intervene during single non-word repetition. In order to answer these questions, studies will need to use experimental designs that allow for the detection of domain-general attention and serial order processing networks instead of factoring them out.

A second prediction is related to sentence repetition. Repeating long sentences with delayed semantic integration, as is for example the case for sentences involving multiple adjectives or subordinate clauses, should put relatively high demands on temporary maintenance processes, and hence should rely on attentional support processes. Martin et al. (2003) showed that sentences where semantic integration is delayed put higher demands on semantic short-term retention abilities. Likewise, verbatim sentence repetition has been shown to be determined by phonological short-term retention abilities (Martin et al., 1999). Therefore, sentence repetition should involve the language repetition pathways as well as the fronto-parietal attention networks involved in short-term maintenance. With respect to the involvement of serial order representation mechanisms, syntactic structure knowledge will on the one hand constrain and determine word order allowing word order to be represented via activation of existing word co-occurrence and syntactic structures in the language network. On the other hand, when this knowledge is not sufficient, as is the case for example for reversible sentence constructions with the two possible interpretations being semantically plausible (e.g., John is being pushed by Eaton vs. Eaton is being pushed by John), the specific coding of word order will be important, potentially needing the recruitment of the serial order representational system supported by the right intraparietal sulcus. In support of this, studies exploring the neural substrates of sentence repetition or generation have shown involvement of both left and right intraparietal sulcus areas (Haller et al., 2005; Tremblay and Small, 2011). In both of these studies, this involvement was even stronger during sentence production than sentence listening/reading: especially sentence production will require detailed attention to both word identity and word order in order to allow for accurate reproduction, while sentence comprehension can be achieved via conceptual level processes for which the retention of specific word order is less determinant, except for the semantically plausible reversible sentence constructions mentioned above. Importantly, Segaert et al. (2013) explored brain activity associated with sentence repetition and observed specific involvement of the right intraparietal sulcus when varying syntactic structure, but not when varying verbs, pointing more directly to a specific role of the right intraparietal sulcus area in supporting processing of syntactic order information; in the same study, the left intraparietal sulcus was involved in the processing of both syntactic structure and verbs, in line with its more general attention processing role.

The role of inhibitory and interference control processes during maintenance of verbal information also has to be briefly discussed. As noted in the first section of this review, the frontal part of the ventral language pathway, i.e., the ventrolateral prefrontal cortex, has been associated with resistance to semantic interference during maintenance of semantic information (Thompson-Schill et al., 2002; Martin et al., 2003; Hamilton and Martin, 2007). A similar mechanism has been proposed for the dorsal language pathway, with the posterior inferior prefrontal cortex associated with phonological interference control processes (Postle, 2005; Schnur et al., 2009; Barde et al., 2010). These studies raise the question of the networks that link these prefrontal phonological and semantic interference control processing areas with the fronto-parietal attention control networks, and this especially in the context of multi-word and sentence processing where there is strong susceptibility for semantic and phonological interference to occur. The results of the dynamic causal modeling study by Makuuchi and Friederici (2013) are informative here since they show that the left inferior parietal cortex is increasingly connected with the inferior frontal cortex (pars opercularis) as a function of the complexity of the sentences to be processed, indicating that the parietal regions involved in attentional control potentially interact with frontal areas supporting inhibitory/interference control processes during sentence processing. Future studies will need to determine the precise task and linguistic conditions in which these interactions between attentional control and interference control networks occur. Furthermore, these processes may also be important to support serial order recall. Hoffman et al. (2012) as well as Jefferies et al. (2008) observed that patients with inhibitory/interference control deficits produced large number of order errors in sentence recall and word list recall. This is also in line with the neuroimaging studies discussed earlier and showing that the network activated when processing serial order information is not limited to the right intraparietal sulcus, but also includes superior frontal and prefrontal areas, including the left inferior prefrontal cortex associated with control of interference/inhibition (Majerus et al., 2006a, 2010). Resolution of interference between items competing for the same serial position is likely to be a further important determining factor of serial order maintenance and recall, especially if word order in a STM lists conflicts with existing word order knowledge structure, as is for example the case when recalling incoherent sentences with words in unexpected sentence positions (Hoffman et al., 2012). This type of process is also often used to model serial order recall in computational models, via competitive cueing and winner-take-all mechanisms (e.g., Burgess and Hitch, 1999).

A further central question relates to the nature of the serial order processing system proposed here and the representations used to represent sequence information. As already noted, this system is supposed to support representation of novel and arbitrary serial order information, with linguistic sequence knowledge as encoded in sublexical, lexical and semantic representations supporting processing of familiar or partially familiar sequences. For the representation of novel, arbitrary order information a number of mechanisms have been proposed, computational models considering that serial order is represented either via episodic context, time-based representations or positional vectors (Gupta and MacWhinney, 1997; Henson, 1998; Brown et al., 2000; Burgess and Hitch, 2006). All these models are able to reproduce the main characteristics of serial position coding such as serial position effects (primacy and recency effects) and transposition gradients during serial recall (items from adjacent positions tend to be exchanged more frequently than items from distant positions). A few models also consider that serial position information may be coded within item representations themselves, by considering that items are represented with different activation levels as a function of serial position or contain rank order information (Page and Norris, 1998; Farrell and Lewandowsky, 2002; Botvinick and Plaut, 2006; Botvinick and Watanabe, 2007). As we have seen, the dissociations observed between item and order processing, at both neuropsychological and neuroimaging levels, are difficult to reconcile with these latter accounts. However, this still leaves the question of the nature of serial order codes open. A possible hypothesis is that the right intraparietal sulcus area involved in serial order coding is involved in the creation of temporary domain-general ordinal representations, allowing the encoding of relational information about items within a sequence, and this in a domain-general manner. This is supported by data showing that this region responds to ordinal information also in other domains such as number processing and alphabetic order processing (Pinel et al., 2001; Fias et al., 2007; Kaufmann et al., 2009; Dormal et al., 2012). The general principle of ordinal coding that is, the assumption that serial order representations vary along a dimension that is organized in some ordinal manner (e.g., ordinal ranks, time-based ordinal information, large-to-small primacy gradient principle) is also at the heart of many of the computational serial order STM models discussed here (Gupta and MacWhinney, 1997; Henson, 1998; Page and Norris, 1998; Brown et al., 2000; Farrell and Lewandowsky, 2002; Botvinick and Plaut, 2006)).

We also cannot exclude the possibility that the right intraparietal sulcus area identified here reflects an ancillary attentional function during the processing of serial order information, given the bilateral intraparietal sulci have been shown to be linked to task-related attention (Todd and Marois, 2004; Duncan, 2010; Majerus et al., 2012). In the studies linking the right intraparietal sulcus to processing and maintenance of serial order information, much care had been taken to equate the item and order STM conditions with respect to task difficulty, which was reflected by equal levels of task performance. However, this does not necessarily guarantee that attentional demands were exactly the same in the two conditions. It may even be the case that a specific form of attentional processes directly supports representation of serial order information. Van Dijck et al. (2013) showed that serial position coding in STM and spatial attention actually interact: they showed a rightward spatial attention bias in a dot detection task which linearly increased as a function of the serial position of the items being retrieved in a concurrent STM task, the bias being largest when items from the end of the STM list were retrieved, and the bias being non-existent when items from the start of the STM list were retrieved. These results give rise to a further hypothesis of serial order coding in STM, namely the involvement of spatial attention and spatial frames (i.e., left-to-right reference frame) as supporting the coding of serial position in STM; this hypothesis is in line with greater involvement of the right vs. left intraparietal sulcus, since the right inferior parietal cortex is known to support this type of attention processes (Bricolo et al., 2002). Finally, as already discussed, executive processes such as control of interference and inhibition supported by ventro-lateral prefrontal cortex are also an important factor associated with serial order maintenance and recall, and in some patients, deficits at this level may explain their serial order deficits (Jefferies et al., 2008; Hoffman et al., 2012). In sum, given the current co-existence of many alternative and not necessarily mutually exclusive hypotheses about the processing of novel, arbitrary serial order information, future studies will be needed to achieve a better understanding of the specific neural and cognitive codes and processes involved in serial order maintenance.

## Conclusions

The account presented here considers that short-term maintenance of verbal information during repetition is not subtended by specific and dedicated storage buffers, contrary to a number of theoretical models of verbal maintenance (Martin et al., 1994b; Baddeley et al., 1998; Baddeley and Logie, 1999; Vallar and Papagno, 2002). Rather, short-term storage results from synchronized and flexible recruitment of language, attentional and serial order processing systems. In this sense, short-term maintenance is an emergent function which depends on neural networks shared with other cognitive functions, including language processing networks (Cowan, 1995; Postle, 2006; Buchsbaum and D'Esposito, 2008). This account is similar to proposals by Postle (2006) and Cowan (1995) who also consider STM as an emergent function, resulting from temporary activation of long-term memory knowledge bases in the language processing networks, and attentional selection and control processes via fronto-parietal networks. Like language processing architectures, these proposals do not specifically consider the role of serial order processing and maintenance. On the other hand, serial order processing has been the focus of very detailed computational frameworks of verbal STM, with some additional consideration for interactions with linguistic representational systems, but no consideration of attentional processes. The present work is an attempt at providing a bridge between three core component processes of verbal short-term maintenance, taking the form of an integrative cognitive and neural framework of the language, attention and serial order processes supporting maintenance during language repetition. This framework provides new perspectives for the understanding of language repetition and maintenance deficits, by allowing for a nuanced and integrative assessment of the multiple components that can lead to breakdown of maintenance of verbal information, including the consideration of the non-linguistic domain-general mechanisms involved in language repetition.

### Conflict of interest statement

The author declares that the research was conducted in the absence of any commercial or financial relationships that could be construed as a potential conflict of interest.

## Footnotes

^1^Note that for the patient described by Hamilton and Martin (2007), the patient's interference control deficits were not limited to semantic information, but also involved phonological information. See page 22 for a discussion of the role of the inferior prefrontal cortex for interference resolution at the semantic versus phonological level.

^2^CG's specific brain lesion could not be clearly determined; a CT scan showed damage to the left anterior lobe; however other white matter lesions could not be excluded due to traumatic brain injury which leads to diffuse axonal injuries which often are not visible via standard CT or MRI scanning protocols (Arfanakis et al., 2002).
